# Easing of Regulatory Barriers to Telemedicine Abortion in Response to COVID-19

**DOI:** 10.3389/fgwh.2021.705611

**Published:** 2021-11-24

**Authors:** Patty Skuster, Jina Dhillon, Jessica Li

**Affiliations:** ^1^Temple University Beasley School of Law, Philadelphia, PA, United States; ^2^Ipas, Chapel Hill, NC, United States; ^3^School of Law, University of California, Berkeley, Berkeley, CA, United States

**Keywords:** abortion, telemedicine, law, policy, COVID-19, medical abortion

## Abstract

For many people seeking abortion during the continuing COVID-19 pandemic, telemedicine abortion is the safest and most acceptable method, posing lower risk of exposure to the virus. In addition, by reducing in-person visits with health care providers, increased use of telemedicine for abortion can reduce pressure on overburdened health systems. Given the benefits of telemedicine during the pandemic, government agencies in several countries took measures to temporarily allow telemedicine abortion. We conducted key-word English-language searches to identify examples of government action to remove regulatory barriers to the practice of telemedicine abortion in response to the pandemic. We found instances of government agencies in eight countries taking steps to ease regulatory barriers to telemedicine abortion. Telemedicine abortion is safe, cost-effective, and may be the preferred method of abortion during acute periods of COVID-19 transmission, as well as after the pandemic has abated. As one step to expanding access to abortion with medicine where abortion is legal, health agencies and other regulatory bodies can take steps to remove barriers specific to telemedicine abortion.

## Background

The COVID-19 pandemic has strained health systems and challenged delivery of health care services, particularly for individuals who live in poverty and face discrimination in other areas of their lives. People who need abortion care have experienced unique difficulties. Abortion is a time-sensitive health service, which is compromised by pandemic-related delays in health care. Disruptions in supply chains and restrictions on travel limit access to both abortion and contraception, and have been expected to increase risk of unwanted pregnancy and the need for abortion care (1). UNPFA, the U.N. sexual and reproductive health agency, estimated that around 47 million women in 114 low and middle-income countries were projected to be unable to use modern contraceptives if a COVID-19 lockdown lasted for 6 months, adding up to two million more for every additional 3 months of lockdown (2).

The pandemic has exacerbated health disparities by multiple measures. Underlying health and social inequities put racial and ethnic minorities at increased risk of getting sick, having more severe illness, and dying (3). Vulnerable groups also are unequally affected by economic and social consequences of COVID-19 mitigation measures (4). Distribution of and access to vaccines has favored richer and whiter populations, at the global and local level (5). Inequities in access to safe abortion care between and within countries are also likely to have widened during the pandemic.

Stigma and politics often override public health and scientific evidence in the formation of abortion law and policy. Abortion is the only type of health care that is in most countries specifically regulated by the criminal law. Generally, national abortion laws criminalize abortion but allow exceptions to criminalization for specific reasons, with robust legal and regulatory requirements that only apply to abortion (6). Anti-abortion policymakers continually erect barriers to abortion. The onset of the pandemic provided a political opportunity for abortion opponents, and a number of governments restricted the provision of health care to services deemed “essential” while explicitly excluding abortion—contrary to evidence that abortion care can be delivered safely and without excessive burden to health care systems (7).

People who live in low-resource settings and those who face discrimination are most impacted by legal and policy barriers to health care. This is certainly true of abortion, as people living in poverty and those who face systematic discrimination by governments lack access to contraception and are more likely to experience unwanted pregnancy and seek abortion, even under unsafe conditions. Abortion is needed by people who face stigma, stereotyping, and discrimination in other aspects of their lives—predominately women, as well as transgender, non-binary, and gender-expansive individuals (8). Globally, rates of unintended pregnancy are higher in poorer countries, and researchers have identified an inverse relationship between unintended pregnancy and income (9).

In response to COVID-19, national governments took steps to increase access to telemedicine for health care services, to overcome some of the challenges related to in-person health service delivery during the pandemic. Telemedicine is the remote assessment and treatment of patients via telecommunications (phone or internet). Telemedicine for health care service delivery was expanding even prior to COVID-19, and can improve the availability, accessibility, and acceptability of health care for people who experience barriers due to poverty, distance from a health care facility, or discrimination (10). Over the course of the COVID-19 pandemic, health providers are able to deliver care via telemedicine while adhering to social distance guidelines and travel-related restrictions while reducing risk of transmission of COVID-19 (11).

Telemedicine is a proven means of providing abortion with medications safely, as health care providers can use telemedicine to provide abortion counseling and assessment, access to abortion medication via pharmacies or mail, and clinical guidance throughout the abortion process (7, 12). WHO-recommended medications for induced abortion are the drugs mifepristone followed by misoprostol or misoprostol alone (13). Both drugs are included in the WHO Model List of Essential Medicines, which means that they should be “available within the context of functioning health systems at all times in adequate amounts, in the appropriate dosage forms, with assured quality, and at a price the individual and the community can afford” (14).

Here we focus on telemedicine abortion (TEMA), as distinct from self-managed abortion, which describes abortion care without the formal involvement of a health care professional (15). While self-managed abortion is also an important means of ending a pregnancy during the pandemic and beyond, laws and policies apply to self-managed abortion differently than they apply to TEMA, and self-managed abortion is therefore beyond the scope of this paper.

This article identifies measures that governments have taken to permit TEMA in response to COVID-19. The changes we describe take place in widely differing contexts and the effect of the policy changes no doubt differs accordingly. Social, economic, cultural, and political factors affect access to abortion, and are beyond the scope of this article. Here we focus on specific changes in the regulatory environment to allow TEMA, to illuminate measures that other governments can take to remove policy barriers to TEMA.

## National-Level Efforts to Improve Access to TEMA

In response to COVID-19, and in a wide variety of contexts, national governments have taken steps to expand telemedicine broadly, by launching virtual platforms or changing licensing requirements (16, 17). But steps to expand telemedicine generally do not necessarily affect the provision of abortion care. People who need abortion services and health workers who provide them are encumbered by a raft of legal and regulatory requirements that go well beyond the requirements of other health care services. Where abortion is permitted, the law generally provides certain conditions under which abortion is legal, which include performance of abortion in specific health care facilities or by specific cadres of health care professionals (18). The dispensing of abortion drugs mifepristone and misoprostol may be restricted to certain health professionals or facilities (19) or stringently regulated, inconsistent with standards set for other drugs (20). To allow telemedicine for abortion care, governments needed to enact measures specific to the regulation of abortion.

### Methods

We identified examples of actions taken by national governments to change the regulatory environment to allow TEMA in response to COVID-19. Given the benefits of TEMA during the pandemic, governments in several countries changed requirements for abortion and, in the case of the United States, announced changes to regulatory enforcement practices to temporarily allow telemedicine abortion.

While the majority of countries regulate abortion exclusively though national law and policy, several including the United States and Germany also regulate abortion through state, provincial, or other sub-national law. To limit the scope of our research, and because of the complexity of U.S. state law in particular, we limited our primary focus to national law.

To identify legal or policy changes in abortion regulation in response to COVID-19, we conducted key-word searches of English-language media reports, SSRN, Google, and PubMed. Our key words included combinations of “COVID-19,” “telemedicine abortion,” “abortion law,” “regulations” and “emergency order.” Once we identified a country where legal changes had taken place, we conducted further searches using the country as a key word to identify additional relevant literature.

### Limitations

Because of the time-intensive nature of researching foreign law, we limited our survey to government action reported in scholarly and gray literature and media reports, and omitted efforts by governments in places that were not examined or reported on by researchers, NGOs, or journalists. Our information is limited to English publications. We also did not investigate actions taken by states, municipalities, provinces, or other sub-national governmental entities. Finally, we recognize that the legal changes we describe are situated in specific country contexts with differing legal traditions and with populations of varied health status. Between countries, legal abortion may be subject to varied levels of stigma and degrees of accessibility, despite being legal in each of the countries discussed. Here, we limited our investigation and analysis to changes in the regulatory environment.

## Results

We identified instances of eight national governments announcing changes in regulation or enforcement (in the U.S.) to ease restrictions on telemedicine abortion in response to the COVID-19 pandemic. In most cases, the changes are temporary. The changes address the location in which two specific steps in the medical abortion process are permitted to take place: (1) consultation and assessment by a health care provider and (2) dispensing of medication. We also describe pre-pandemic regulatory changes in the United Kingdom which address a third step in the medication abortion process: (3) where the second abortion medication is permitted to be ingested. If the regulatory framework requires people who need abortion to complete one, two, or all three steps in-person at a formal health care facility, entirely remote provision of abortion care remains prohibited.

### Permitted Remote Consultation and Assessment by an Abortion Provider

The regulated action that was subject to change in the largest number of countries we identified was consultation and assessment by a health care provider. The governments of England, Scotland, Wales, Ireland, France, Germany, and South Africa all made changes to the regulatory framework to allow remote rather than an in-person visit to a health facility.

Ireland's Regulation of Termination of Pregnancy Act of 2018 required two in-person consultations with a medical practitioner, separated by a mandatory 3-day waiting period (21, XXX). In April 2020, Ireland's Department of Health issued temporary emergency provisions, intended to only last for the duration of the COVID-19 pandemic, which allow the two mandatory consultations for abortion to take place remotely (21). Medical practitioners may choose to require that at least one of the consultations occur in-person, but the provisions recommend that in-person consultations be kept to a minimum during the COVID-19 public health emergency (21–23).

In March 2020 in France, the Minister of Solidarity and Health made an urgent request to the French National Health agency to address the need for abortion during the pandemic. In response, the French National Health Agency issued an emergency order (24–26). In addition to measures governing drug dispensing (addressed below), the order permitted remote consultation for TEMA within 63 days of amenorrhea for as long as the COVID-19 pandemic is ongoing (25, 26).

In Germany, where TEMA remains prohibited and abortion care must be administered in a clinical setting, the Government permitted telemedicine for the mandatory pre-abortion counseling that is provided by an independent third party as required by the German Criminal Code (24, 27–29).

Unique in our research, in South Africa changes were made to the provision of telemedicine broadly and those changes also applied to telemedicine for abortion. TEMA had been permitted prior to the pandemic. However, telemedicine was only permissible where an already established practitioner-patient relationship existed. This was changed in response to COVID-19 in March 2020, when the Health Professions Council of South Africa (a statutory body) amended its telemedicine guidelines to permit telehealth without a prior relationship “provided that such consultations are done in the best clinical interest of patients” (30, 31).

The three Health Agencies governing England, Scotland, and Wales all issued orders to allow remote consultation and prescription for abortion drugs. On March 30, 2020 The UK Department of Health and Social Care issued an emergency order to permit pregnant women to be prescribed abortion medication by video link, telephone, or any other means (32, 33). On March 31, 2020, Scotland and Wales issued similar approval orders, to also allow remote clinic visits (32, 34, 35).

The UK and Welsh orders are due to expire in March 2022 or when the temporary provisions in the Coronavirus Act 2020 expire—whichever date is earlier. While the approval order for Scotland does not include an expiry date, it is also intended as a temporary measure. In a letter, the Scottish Chief Medical Officer explained that,

[They] intend that it will have effect for a limited period and so would revoke it and replace it with the terms of the previous approval (dated October 2017) at an appropriate time when it is judged that it is no longer necessary in relation to the pandemic response (35).

### Permitted Dispensing of Abortion Drugs by Pharmacy or Mail

Government agencies in the United States and France both changed requirements of where a pregnant person can acquire medication abortion, in effect allowing medication to be dispensed in pharmacies or by mail.

In the United States abortion services are primarily regulated by state governments. Early in the pandemic 19 state governments took measures to suspend abortion services in response to the COVID-19 emergency (36). While some state officials in the US relaxed restrictions on telemedicine more generally, several also excluded abortion from their telemedicine policies (37).

At the national level, the Federal Drug Administration (FDA) regulates mifepristone under the FDA's risk evaluation and mitigation strategies (REMS) protocol (38) despite clinical evidence that the safety of mifepristone does not require such treatment. Under REMS, mifepristone must be dispensed from provider to patient in-person in a clinic, hospital, or other medical setting so even though the abortion occurs at the patient's home, they cannot obtain abortion drugs from the pharmacy or receive them by mail (39). In April 2021, the FDA moved to expand access to telemedicine abortion when it signaled that it would not enforce in-person dispensing requirements for abortion drugs during the pandemic (40).

The March 2020 emergency order by the French National Health Agency to address the need for abortion during the pandemic also permits pregnant people to obtain abortion medication from a pharmacy, temporarily easing requirements that required medication for abortion to be obtained only from a doctor or midwife.

### Permitted Ingestion of Medication at Home

While Scottish, Welsh, and UK health authorities permitted remote clinic visits via emergency orders in March 2020, all three agencies had already allowed pregnant women to self-administer misoprostol at home. Between 2017 and 2018, health officials for the UK, Scottish, and Welsh governments issued orders to allow pregnant people to take abortion drugs at home if they had attended a clinic to be prescribed both mifepristone and misoprostol, been supervised administering mifepristone in the clinic, and were ordinarily a resident at the place where they self-administer misoprostol (36, 41–43). Because women would not be legally permitted to obtain TEMA without an in-person visit to a clinic prior to March 2020, the emergency orders allowing remote visits were necessary for TEMA to be permitted.

## Discussion

The COVID-19 pandemic has spotlighted deficiencies in health law and policy across the globe, not least in the regulation of abortion. The need for governments to address abortion separately from other health care services in response to the pandemic reinforces the arbitrary nature of abortion law and policy. Telemedicine abortion is simple and safe, yet separate provisions govern the specific location of each step in the process, without health or safety justification.

Health officials eased regulatory barriers to telemedicine abortion in response to the pandemic, but they did so on a temporary basis. Governments lack a basis to re-impose requirements that prohibit telemedicine abortion. After the risk of transmission of COVID-19 has abated, telemedicine will remain a safe and preferred method of abortion for some pregnant people and should continue to be allowed.

An evidence-based approach to regulation of abortion includes removal of abortion from the criminal law and the end of arbitrary legal and regulatory barriers to all modes of abortion care—including in-clinic, telemedicine, and self-managed abortion.

Where Ministries of Health and other health officials are limited by the legal and regulatory environment in their efforts to improve access to abortion, there may be measures they can take to reduce regulatory burdens. Health ministries may have potential avenues to address policies that govern where people seeking abortion are counseled, obtain abortion medicines, and ingest them, to allow every step in the process of abortion with medicine to take place at home, or wherever the pregnant person chooses. Through specific guidance or exercise of enforcement discretion, government officials may be able to permit telemedicine to better meet the needs of individuals who need abortion care.

## Data Availability Statement

The original contributions presented in the study are included in the article/supplementary material, further inquiries can be directed to the corresponding author.

## Author Contributions

PS came up with the concept of the research and JD added ideas for framing. JL executed legal research. All authors contributed to the article and approved the submitted version.

## Conflict of Interest

The authors declare that the research was conducted in the absence of any commercial or financial relationships that could be construed as a potential conflict of interest.

## Publisher's Note

All claims expressed in this article are solely those of the authors and do not necessarily represent those of their affiliated organizations, or those of the publisher, the editors and the reviewers. Any product that may be evaluated in this article, or claim that may be made by its manufacturer, is not guaranteed or endorsed by the publisher.
